# Hyaluronate/black phosphorus complexes as a copper chelating agent for Wilson disease treatment

**DOI:** 10.1186/s40824-021-00221-x

**Published:** 2021-06-16

**Authors:** Seong-Jong Kim, Hye Hyeon Han, Sei Kwang Hahn

**Affiliations:** Department of Materials Science and Engineering, Pohang University of Science and Technology (POSTECH), 77 Cheongam-ro, Nam-gu, Pohang, Gyeongbuk 37673 South Korea

**Keywords:** Black phosphorus nanosheets, Hyaluronate, Chelating agent, Wilson disease

## Abstract

**Background:**

Wilson disease (WD) is a genetic disorder of copper storage, resulting in pathological accumulation of copper in the body. Because symptoms are generally related to the liver, chelating agents capable of capturing excess copper ions after targeted delivery to the liver are highly required for the treatment of WD.

**Methods:**

We developed hyaluronate-diaminohexane/black phosphorus (HA-DAH/BP) complexes for capturing copper ions accumulated in the liver for the treatment of WD.

**Results:**

HA-DAH/BP complexes showed high hepatocyte-specific targeting efficiency, selective copper capturing capacity, excellent biocompatibility, and biodegradability. HA enhanced the stability of BP nanosheets and increased copper binding capacity. In vitro cellular uptake and competitive binding tests verified targeted delivery of HA-DAH/BP complexes to liver cells via HA receptor mediated endocytosis. The cell viability test confirmed the high biocompatibility of HA-DAH/BP complexes.

**Conclusion:**

HA-DAH/BP complexes would be an efficient copper chelating agent to remove accumulated copper in the liver for the WD treatment.

## Background

Wilson disease (WD) is an inborn disorder of copper metabolism and characterized by copper overload in the organs, especially liver and brain [1]. In patients with WD, copper cannot be eliminated properly, causing liver cirrhosis and liver transplantation in severe cases [2]. Accordingly, WD is one of the most challenging diseases in medicine. There are two available treatments to increase copper excretion and reduce copper absorption by using (1) chelators and (2) zinc salts [3]. Combination therapy using zinc salts and chelators leads to clearing copper overloaded tissues and blocking the pathological accumulation of copper in the organs [4, 5]. However, the long-term use of the medication is limited because of the safety issues and severe adverse effects [6]. Chelators, such as D-penicillamine and trientine, have been reported to cause adverse events like marrow toxicity, lupus-like syndrome, and anemia [7, 8]. As a suitable chelator, the agent should be able to efficiently capture excess transition-metal ions with excellent biocompatibility and biodegradability. Recently, various nanomaterials have been widely investigated as novel chelators to form nontoxic metal complexes [9]. In addition, nanoparticle-mediated drug delivery efficiently reduced several toxic metals in the body [10]. However, there are few in-depth studies focusing on targeted delivery of chelating agents to the liver for the treatment of WD.

Here, we developed a hyaluronate-diaminohexane/black phosphorus (HA-DAH/BP) complex to remove accumulated copper, especially in the liver. BP nanosheets are well known as a biodegradable 2D material composed of phosphorus atoms [11, 12]. Due to its great biocompatibility, BP nanosheets have been used for biomedical applications including phototherapy, drug delivery, and biocatalysis [13]. Furthermore, phosphorus binds strongly with metal ions, especially Cu^2+^, making BP nanosheets a robust nanocaptor for copper ions [9]. To prevent the rapid degradation of BP nanosheets from oxidation, BP nanosheets were coated with HA [14]. HA is a natural biodegradable polymer with a high binding affinity toward liver cells [15–18]. After the physicochemical characterization of HA-DAH/BP complexes, we investigated the copper capturing capability, biocompatibility and biodegradability via in vitro liver-specific targeted delivery of HA-DAH/BP complexes for WD treatment.

## Methods

### Materials

Bulk crystals of black phosphorus (BP) were obtained from Smart-elements (Vienna, Austria). Sodium hyaluronate (HA, MW = 10 kDa) was acquired from Lifecore Biomedical (Chaska, MN, USA). 1-Methyl-2-pyrrolidinone (NMP), 1,6-diaminohexane (DAH), dimethyl sulfoxide (DMSO), and N-hydroxysulfosuccinimide (NHS) sodium salt were purchased from Sigma Aldrich (St. Louis, MO). 5-Aminofluorescein (FITC) and 1-ethyl-3-(3-(dimethylamino)propyl) carbodiimide (EDC) hydrochloride were purchased from Tokyo Chemical Industry (Tokyo, Japan). Cell counting kit-8 (CCK-8) was acquired from Dojindo Molecular Technologies (Kumamoto, Japan). Dulbecco’s modified Eagle’s medium (DMEM), fetal bovine serum (FBS), and antibiotics were purchased from Gibco (Grand Island, NY). HepG2 cells were purchased from Korean Cell Line Bank (Seoul, Korea).

### Preparation of BP nanosheets

BP nanosheets were prepared by liquid exfoliation technique. In brief, 30 mg of BP was dispersed in 30 mL NMP solution with sodium hydroxide. Then, the solution was sonicated for 10 h (amplifier: 20%, on/off: 6 s / 4 s). The non-exfoliated BP nanosheets were removed using centrifugation for 10 min at 5000 rpm and the supernatant was centrifuged again for 15 min at 12,500 rpm to re-disperse BP nanosheets in distilled (DI) water.

### Preparation of HA-DAH/BP complexes

HA-DAH was synthesized by the EDC chemistry between HA (10 kDa, 100 mg) and DAH (578 mg, 10 M ratios to HA) in DI water at pH 4.8 for 24 h. Then, HA-DAH was dialyzed against 15% ethanol and DI water, respectively (MWCO = 3500 Da). HA-DAH was freeze-dried for 3 days. After that, BP nanosheets were mixed with HA-DAH at an equal content and stirred for 1 h to form HA-DAH/BP complexes. The solution was centrifuged for 15 min at 12,500 rpm to remove excess HA-DAH and obtained HA-DAH/BP complexes were dispersed in DI water.

### Characterization of BP nanosheets and HA-DAH/BP complexes

The prepared BP nanosheets and HA-DAH/BP complexes were analyzed by dynamic light scattering (DLS, Zetasizer Nano ZS90, Malvern Instruments Co., Malvern, UK), UV/vis spectrophotometry (S-3100, Scinco Co., Seoul, Korea), Fourier transform - infrared spectroscopy (FT-IR, Cary 600, Agilent Technologies), and transmission electron microscopy (TEM, JEM-1011, JEOL Co., Akishima, Japan). The physical structure of BP nanosheets and HA-DAH/BP complexes was analyzed by TEM, and the surface modification of BP nanosheets with HA was assessed by DLS and FT-IR.

### Stability and biodegradation tests

BP nanosheets and HA-DAH/BP complexes were suspended in DI water at an equal concentration and then the absorbance spectrum of each solution was measured for 7 days by UV/vis spectrophotometry.

### In vitro release test of FITC

First, HA-FITC conjugates were synthesized by the EDC chemistry between FITC and HA-DAH with a DAH content of 50 mol%. FITC/BP complexes were prepared by simple mixing of BP nanosheets and FITC, and HA-FITC/BP complexes were also prepared by simple mixing of BP nanosheets and HA-FITC conjugates via electrostatic interaction. Solutions were centrifuged for 15 min at 12,500 rpm to remove excess FITC or HA-FITC. After 0, 48, 96, and 144 h, fluorescent intensity of FITC/BP and HA-FITC/BP complexes was analyzed using a microplate fluorometer (Fluoroskan ascent FL, Thermo Scientific). The released amount of FITC was calculated from the decrease of the fluorescent intensity.

### Determination of cu(Cu) content

CuSO_4_ aqueous solution (10 mL, 40 μM) was mixed with BP nanosheets or HA-DAH/BP complexes solution (10 mL, 20 μg mL^− 1^) for 1 h. BP nanosheets and HA-DAH/BP complexes were prepared at the desired degradation time point from day 0 to 6. After centrifugation for 15 min at 12,500 rpm, the concentration of metal ions in the supernatants was measured using an atomic absorption spectrometer (TAS-990, Puxi, China).

### Binding capacity of BP nanosheets and HA-DAH/BP complexes to metal ions

Each metal ion stock solution was prepared from CaCl_2_, Mg (NO_3_)_2_, ZnCl_2_, FeCl_3_·6H_2_O and CuSO_4_·5H_2_O, respectively. BP nanosheets or HA-DAH/BP complexes solution (10 mL, 20 μg mL^− 1^) was mixed with 10 mL of the metal ion stock solution for 1 h. After centrifugation for 15 min at 12,500 rpm, the concentration of metal ions in the supernatants was measured using an atomic absorption spectrometer (TAS-990, Puxi, China). The binding capacity was calculated by the following equation as previously reported elsewhere [9]:

$$ \mathrm{Binding}\ \mathrm{capacity}=\frac{{\mathrm{C}}_{\mathrm{T}}-{\mathrm{C}}_{\mathrm{s}}}{{\mathrm{C}}_{\mathrm{T}}}\times 100 $$

where *C*_*T*_ represents the total concentration of metal ions in the mixture and *Cs* represents the concentration of metal ions in the supernatant.

### Characterization of BP-Cu and HA-DAH/BP-Cu complexes

The energy dispersive spectroscopy mapping (EDS mapping) of BP, BP-Cu, HA-DAH/BP, HA-DAH/BP-Cu was performed with a field emission - scanning electron microscope (FE-SEM, JSM-7401F, JEOL, Akishima, Japan).

### In vitro biocompatibility test

Liver cancer cells, HepG2, in DMEM were maintained in a humidified 5% CO_2_ incubator at 37 °C. HepG2 cells were seeded at a density of 2 × 10^4^ onto 96-well plates and treated with different concentrations of BP nanosheets and HA-DAH/BP complexes for 24 h. Serum-free medium and CCK-8 were added to the cells after washing with PBS. The relative cell viability (*n* = 4) was assessed by the microplate reader (EMax microplate reader, Bucher Biotec AG, Basel, Switzerland).

### In vitro cellular uptake

HepG2 cells were seeded at a density of 1 × 10^4^ cells onto the confocal dish. After incubating for a day, the cells were pre-incubated with excess HA for 2 h to confirm HA receptor-mediated endocytosis of HA-FITC/BP complexes. FITC/BP or HA-FITC/BP complexes were added, incubated for 2 h, and washed with PBS. The cells were fixed with 4% paraformaldehyde solution and stained with DAPI. Images were acquired by confocal microscopy (TCS SP5 Ltd., Leica Korea).

### Statistical analysis

Statistical comparison was performed using the software SigmaPlot 10.0 (Systat Software Inc. San Jose, CA). Values for *P < 0.05 and ***P < 0.001 were considered significant.

## Results

### Preparation and characterization of HA-DAH/BP complexes

**Fig. 1:**
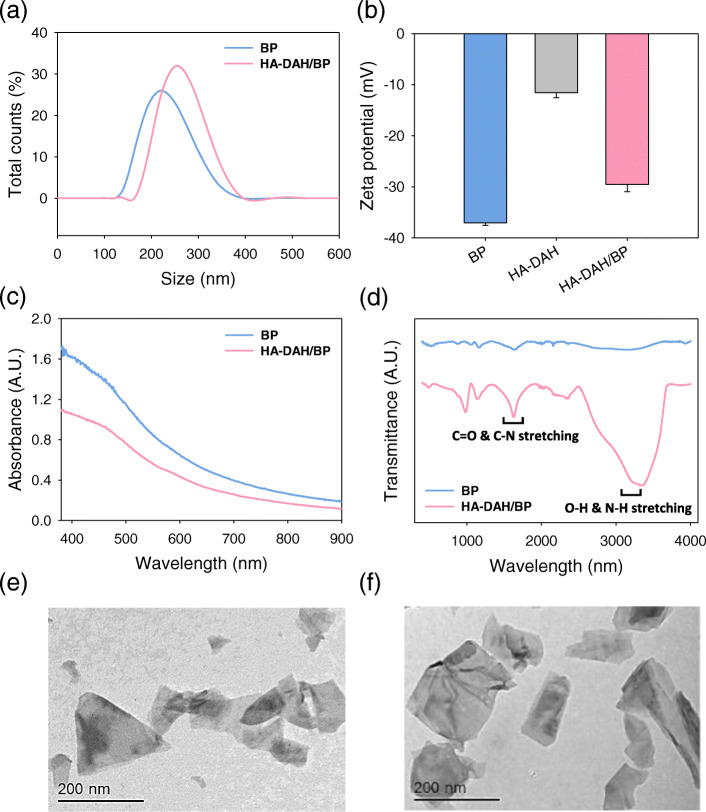
Characteristics of BP nanosheets and HA-DAH/BP complexes. **a** Size distribution, **b** zeta potential, **c** absorbance spectra and **d** FT-IR spectra of BP nanosheets and HA-DAH/BP complexes. TEM images of **e** BP nanosheets and **f** HA-DAH/BP complexes. Data are expressed as mean ± SD

BP nanosheets were prepared from bulk BP by liquid exfoliation. Then, HA-DAH/BP complexes were synthesized by simple mixing of BP nanosheets with HA-DAH. BP nanosheets and the amino groups of HA-DAH were physically bound by electrostatic interaction. DLS revealed the size increase from 226.6 ± 46.6 nm to 258.8 ± 42.3 nm after mixing of BP nanosheets with HA-DAH (Fig. 1a). The zeta potential of HA-DAH/BP complexes (− 29.6 ± 1.1 mV) was increased from − 37.1 ± 0.4 mV due to amino groups of HA-DAH (Fig. 1b). There was no obvious change in the absorbance spectra of BP nanosheets after HA coating (Fig. 1c). To confirm the successful synthesis of HA-DAH/BP complexes, FT-IR spectroscopy was performed (Fig. 1d). FT-IR spectrum of HA-DAH/BP complexes showed noticeably increased peaks of O-H & N-H stretching (3000–3500 cm^− 1^) and amide C=O & C-N stretching (1600–1700 cm^− 1^). TEM showed that both BP nanosheets and HA-DAH/BP complexes were free-standing with a uniform planar morphology (Fig. 1e and f).

### Enhanced stability and biodegradation of HA-DAH/BP complexes

**Fig. 2:**
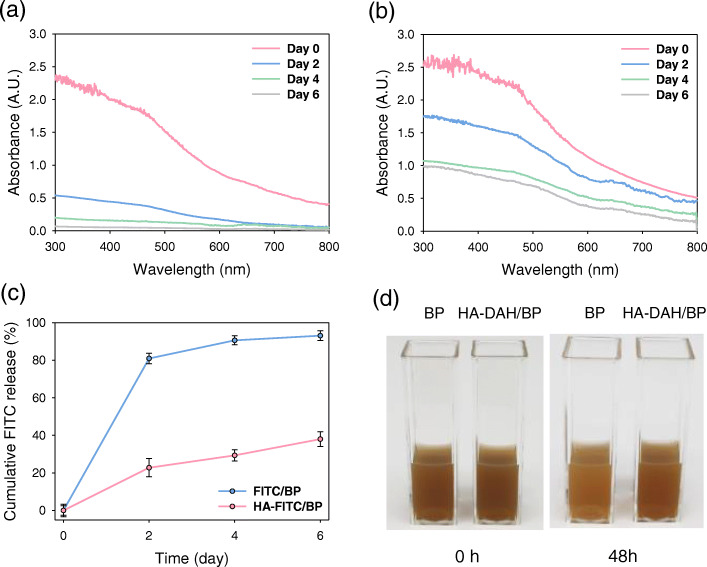
Stability of HA-DAH/BP complexes. Absorbance spectra of **a** BP nanosheets and **b** HA-DAH/BP complexes in DI water for 7 days. **c** In vitro release of FITC from FITC/BP and HA-FITC/BP complexes in DI water for a week (*n* = 3). **d** Photographs of BP nanosheets and HA-DAH/BP complexes in DI water for 48 h

BP nanosheets are known to be decomposed into phosphoric acid (PA) in the presence of oxygen [19]. HA can prevent the rapid degradation of BP nanosheets under oxygen circumstance and aggregation with the adsorption of serum proteins [20, 21]. To assess the effect of HA on the stability of BP nanosheets, BP nanosheets and HA-DAH/BP complexes at the equal concentration were dispersed in DI water. As shown in Fig. 2a, the absorbance of BP nanosheets was significantly decreased for 7 days and barely remained at day 6. In contrast, HA-DAH/BP complexes were stable without precipitation and the absorbance spectra was nearly unchanged after 4 days (Fig. 2b). In addition, the enhanced stability of HA-DAH/BP complexes was assessed by the fluorescent intensity of FITC (Fig. 2c). In vitro release of FITC indicated that FITC or HA-FITC was separated from FITC/BP or HA-FITC/BP complexes by degradation. The amount of FITC released from HA-FITC/BP complexes was relatively small with improved stability, and the concentration of FITC was consistent with the degradation rate (Fig. 2a and b). The colour of BP nanosheets solution became thin after 7 days, compared to that of HA-DAH/BP complexes solution (Fig. 2d). All these results confirmed that HA efficiently protected BP nanosheets from subsequent degradation.

### Copper specific capturing of HA-DAH/BP complexes

**Fig. 3:**
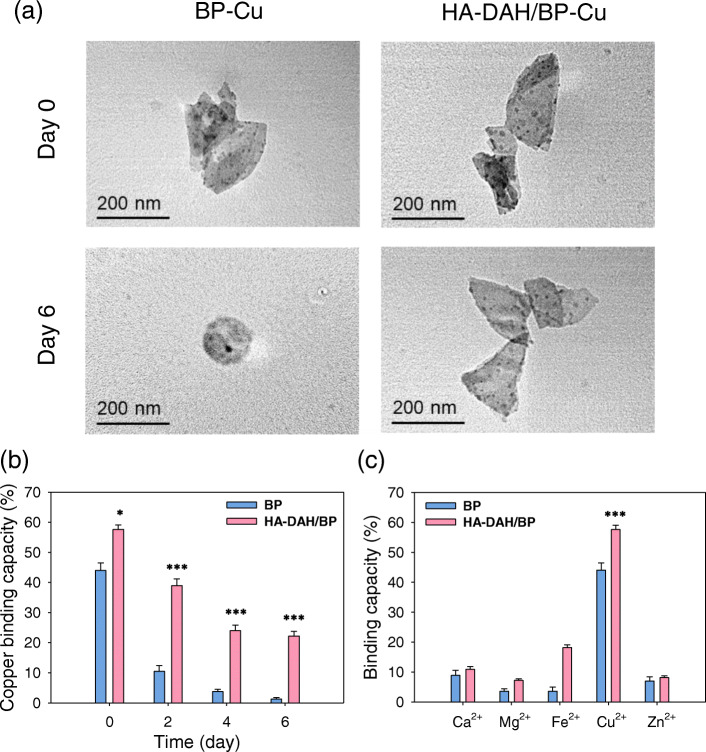
Copper capturing capacity of HA-DAH/BP complexes. **a** TEM images of BP-Cu and HA-DAH/BP-Cu complexes after degradation of BP nanosheets and HA-DAH/BP complexes for 7 days. The binding capacity of BP nanosheets and HA-DAH/BP complexes to **b** copper ions after degradation of BP nanosheets and HA-DAH/BP complexes for 7 days (*n* = 3, *P < 0.05, ***P < 0.001, HA-DAH/BP complexes versus BP nanosheets) and **c** different metal ions after mixing for 1 h (*n* = 3, ***P < 0.001, copper ion versus other metal ions for HA-DAH/BP complexes)

The Cu^2+^ capturing capacity of BP nanosheets and HA-DAH/BP complexes was investigated by mixing their solutions with Cu^2+^ solution for 1 h, centrifugation, and dispersion in DI water. The sub-band of 129.3 eV confirms the formation of BP-Cu complexes in the XPS spectrum [9]. TEM images showed that BP nanosheets and HA-DAH/BP complexes maintained their structures after binding with Cu^2+^ (Fig. 3a). After 7 days, the degraded BP nanosheets and the degraded HA-DAH/BP complexes interacted with Cu^2+^. TEM images showed that only HA-DAH/BP complexes still maintained the nanosheets structure binding with Cu^2+^ (Fig. 3a). The morphology of BP nanosheets disappeared and the spherical shape was obtained only due to copper [22]. To evaluate the effect of degradation on copper binding capacity, BP nanosheets and HA-DAH/BP complexes were prepared at the desired degradation time point from day 0 to 6. After mixing the solution of BP nanosheets or HA-DAH/BP complexes with Cu^2+^ solution for 1 h, the mixture was centrifuged and the concentration of Cu^2+^ in supernatants was analyzed with an atomic absorption spectrometer. As shown in Fig. 3b, the Cu^2+^ capturing capacity of BP nanosheets was dramatically decreased for 7 days. In contrast, the copper binding capacity of HA-DAH/BP complexes was only slightly decreased owing to the HA coating, which was well matched with the degradation rate of HA-DAH/BP complexes in Fig. 2b.

Meanwhile, the copper specific binding of BP nanosheets and HA-DAH/BP complexes was assessed with an atomic absorption spectrometer by comparing the intensity change of each metal ion before and after mixing for 1 h (Fig. 3c). BP nanosheets and HA-DAH/BP complexes selectively interacted with Cu^2+^, which indicated the excellent copper capturing specificity. The concentration of other metal ions, such as Ca^2+^, Mg^2+^, and Zn^2+^ was nearly unchanged. The reason might be that the Gibbs free energy change (ΔG) of BP-Cu or HA-DAH/BP-Cu complexes was much lower than that of BP-metal or HA-DAH/BP-metal complexes [9]. The binding capacity of HA-DAH/BP complexes to Cu^2+^ and Fe^2+^ was slightly increased due to the complex formation [23, 24].

**Fig. 4:**
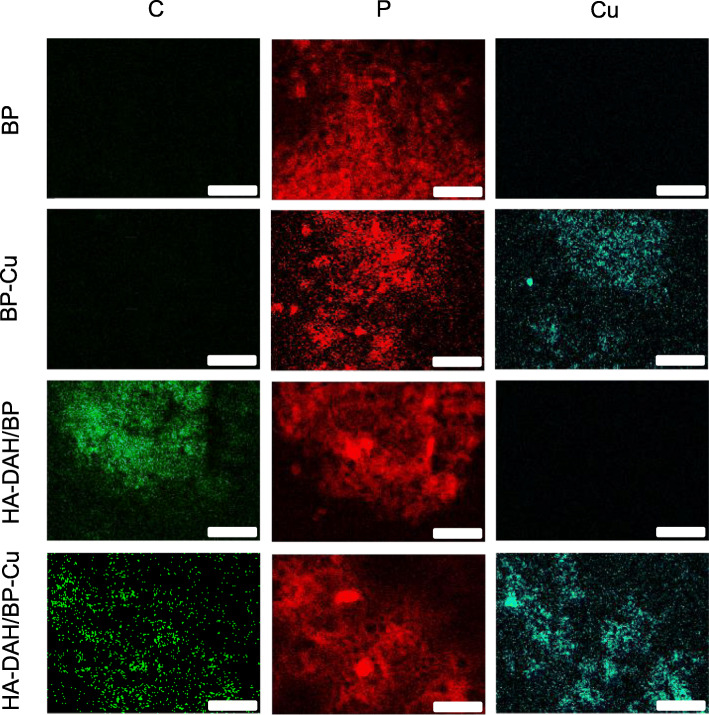
FE-SEM elemental mapping of C, P, and Cu for BP nanosheets, BP-Cu, HA-DAH/BP, and HA-DAH/BP-Cu complexes (scale bar = 2.5 μm)

We next investigated the distribution of copper on BP nanosheets and HA-DAH/BP complexes by the FE-SEM element mapping (Fig. 4). The C element EDS mapping was only observed in HA-DAH/BP complexes because of HA carbon chains. Copper ions were co-localized with P elements on the EDS mapping of BP-Cu and HA-DAH/BP-Cu complexes. These results successfully demonstrated the high capturing capacity of HA-DAH/BP complexes to Cu^2+^.

### In vitro cellular uptake and biocompatibility

**Fig. 5:**
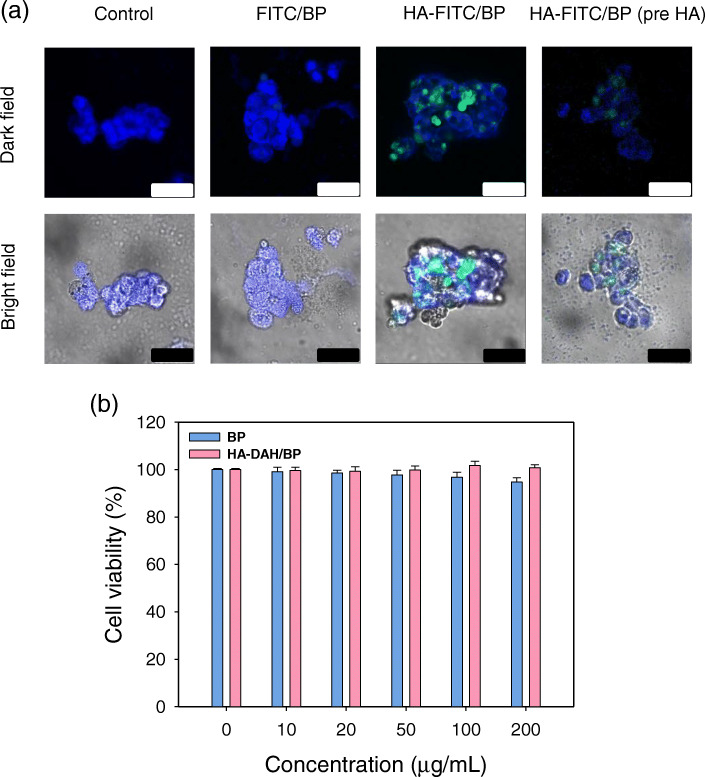
Cellular uptake and biocompatibility assessment of HA-DAH/BP complexes. **a** Confocal microscopy of HepG2 cells after treatment with FITC/BP and HA-FITC/BP complexes (scale bar = 50 μm) without and with HA pre-incubation (Blue: DAPI and Green: FITC). **b** The biocompatibility of BP nanosheets and HA-DAH/BP complexes in HepG2 cells by the CCK-8 assay (*n* = 4). Data are expressed as mean ± SD

The cellular uptake of FITC/BP and HA-FITC/BP complexes into the liver cancer cells, HepG2, was investigated by confocal microscopy. HA-FITC/BP complexes only showed green fluorescence of FITC, indicating the liver cell targeted delivery of HA-DAH/BP complexes (Fig. 5a). HA interacts with liver cells via HA-binding receptors, such as receptor for HA-mediated motility (RHAMM) and CD44 [20, 25]. To study the cellular uptake mechanism of HA-FITC/BP complexes, HepG2 cells were pre-treated with excess HA. The fluorescence of HA-FITC/BP complexes was reduced in the cells pre-incubated with HA, which revealed that the uptake of HA-FITC/BP complexes was mediated by HA receptor-mediated endocytosis. The in vitro biocompatibility of BP nanosheets and HA-DAH/BP complexes was analyzed by CCK-8 assay. As shown in Fig. 5b, the viability of HepG2 cells after treatment with HA-DAH/BP complexes was slightly higher than that with BP nanosheets, which might be ascribed to the biocompatible HA coating [26, 27].

## Discussion

**Fig. 6:**
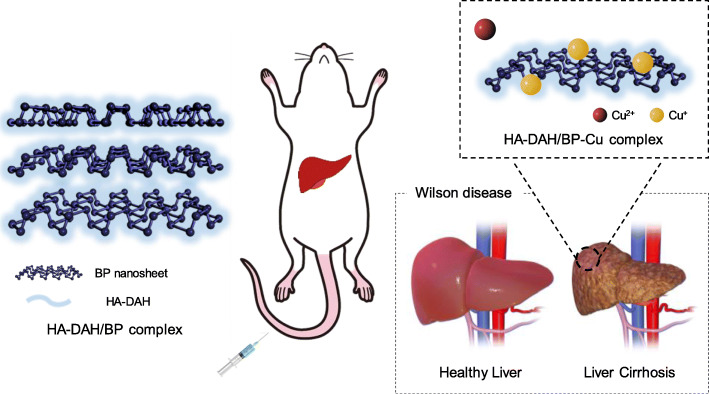
Schematic illustration of HA-DAH/BP complexes as a copper chelating agent to remove copper ions accumulated in the liver for WD treatment

This study aimed to protect BP nanosheets from rapid degradation in oxygen circumstances and increase copper binding capacity for WD treatment. Previous studies have reported limitations of BP nanosheets in biomedical applications due to the rapid degradation of BP nanosheets [13, 19]. As shown in Figs. 2 and 3, BP nanosheets were rapidly decomposed into phosphorus atoms and copper binding capacity also decreased with the degree of decomposition. To overcome this issue, BP nanosheets have been coated with various polymers such as polyethylene glycol (PEG), HA, and poly lactic-co-glycolic acid (PLGA) [28, 29]. However, there are no previous studies of the change in the copper binding capacity of BP nanosheets after surface modification. Also, although the liver is the major organ where excess copper accumulates in WD, there are few in-depth studies on delivering chelating agents to the liver for the treatment of WD. In this research, BP nanosheets were modified with HA for improved stability and targeted delivery to the liver. HA has been widely investigated in the biomedical field due to its excellent biocompatibility and targeted delivery to the liver or tumor [17, 18, 20]. Our results demonstrated that HA successfully prevented BP nanosheets from rapid degradation and maintained the Cu^2+^ capturing capacity of BP nanosheets after degradation for 7 days (Figs. 2 and 3). The most significant implication was that the copper binding capacity of HA-DAH/BP complexes was higher than that of BP nanosheets, as shown in Fig. 3b and c. The complex formation between HA-COO^−^ and copper ions increases the copper binding capacity of HA-DAH/BP complexes [24, 30]. Moreover, the cellular uptake of HA-DAH/BP complexes into the liver cancer cells, HepG2, demonstrated the liver cell targeted delivery of HA-DAH/BP complexes (Fig. 5a). Our results sufficiently showed enhanced stability, copper-specific binding capacity, and hepatic targeted delivery of HA-DAH/BP complexes. This study could be further processed for in vivo animal studies of WD treatment, as schematically shown in Fig. 6.

## Conclusion

We have successfully developed HA-DAH/BP complexes as a copper specific chelating agent for the treatment of WD. HA-DAH/BP complexes could selectively capture Cu^2+^ among various transition-metal ions, such as Ca^2+^, Mg^2+^, Fe^2+^, and Zn^2+^. The copper binding capacity of HA-DAH/BP complexes was significantly higher than that of BP nanosheets. In addition, HA was able to prevent BP nanosheets from rapid degradation under oxygen environment and maintain the Cu^2+^ capturing capacity of HA-DAH/BP complexes. With enhanced stability, HA-DAH/BP complexes showed excellent biocompatibility in HepG2 cells. In vitro cellular uptake test with HepG2 cells confirmed the feasibility for targeted delivery of HA-DAH/BP complexes to the liver. Taken together, HA-DAH/BP complexes would be a promising liver targeting and copper chelating agent for WD treatment.

## Data Availability

All data analyzed in this study are included in this published article.
